# Clinical Significance of miR-149 in the Survival of Patients with Laryngeal Squamous Cell Carcinoma

**DOI:** 10.1155/2016/8561251

**Published:** 2016-06-15

**Authors:** Yi Xu, Yun-Peng Lin, Dong Yang, Geng Zhang, Hui-Fang Zhou

**Affiliations:** ^1^Department of Otorhinolaryngology, Tianjin Medical University General Hospital, Tianjin 300052, China; ^2^Department of Neurosurgery, Tianjin Medical University General Hospital, Tianjin 300052, China

## Abstract

MicroRNAs (miRNAs) play critical roles in the progression of laryngeal cancer (LC). In this study, we aimed to investigate whether miR-149 is associated with the prognosis of patients with LC. A total of 97 laryngeal squamous cell carcinoma patients who underwent tumor resection were included in our follow-up study.* In vitro* studies was performed in cancer cell line Hep-2 to explore the antitumor role of miR-149 in LC. We found that the expression of miR-149 was significantly lower in tumor tissues, compared with vocal cord polyp tissues (*P* < 0.05). Kaplan-Meier analysis revealed that miR-149 expression status is significantly associated with survival duration (log rank test, *P* < 0.05), and multivariate Cox regression analysis revealed that patients with low miR-149 expression had shorter survival times compared with patients with high miR-149 expression.* In vitro* studies revealed that the exogenous expression of miRNA-149 inhibits the proliferation of human Hep-2 cells and induces cell apoptosis. Our study suggests that miR-149 expression in laryngeal squamous cell carcinoma tissues is critically associated with the prognosis of patients, and the ectopic expression of miR-149 in Hep-2 cells inhibits proliferation and cell cycle progression.

## 1. Introduction

Laryngeal cancer (LC) is one of the most common malignant tumors that occur in the head and neck, and the vast majority of tissue types of LC are laryngeal squamous cell carcinoma (LSCC) [1, 2]. It has been reported that the incidence of laryngeal cancer is approximately 0.01–0.03% [3, 4]. With the development of modern industries and the aggravation of air pollution, the incidence of LC has gradually increased [5]. The north and east regions have the highest incidence of LC in China. Currently, the etiology of laryngeal carcinoma is not clearly elucidated. It is generally believed that smoking, alcohol drinking, exposure to harmful dust, and HPV infection are related to the occurrence of LC [6]. As a factor, the occurrence and development of LC are a complex process involved in multiple genes and pathways. The development and metastasis of LC are closely related to the primary site, differentiation, and tumor size.

Surgery and radiotherapy are the main treatment methods for LC. With the development of modern technology, the survival rate of LC patients has improved in recent years. However, the early diagnosis of LC has had little progress and further studies are need. In recent years, compelling researches have indicated that miRNA is involved in the biological processes of many tumors including growth, metabolism, cell differentiation, proliferation, and apoptosis [7–9].

The downregulation of miR-149, a small noncoding RNA, has been reported in non-small-cell lung cancer (NSCLC) [10], glioma, and gastric cancer [11, 12]; and miR-149 functioned as a tumor suppressor in humans. Although Luo et al. mentioned that miR-149 promotes epithelial-mesenchymal transition and invasion in nasopharyngeal carcinoma cells [13], Wang et al.'s research revealed that the ectopic expression of miR-149 in gastric cancer cells inhibits proliferation and cell cycle progression [14]. Furthermore, Zhan et al. reported that the downregulation of miRNA-149 decreased the sensitivity of ovarian cancer A2780 cells to paclitaxel [15].

According to the above reported literatures, we speculated that miRNA-149 has a similar expression difference in LC and a changing biological behavior in the cell level. In this study, we investigated the expression of miRNA-149 in LC and vocal cord polyp tissues and investigated the biological function and clinical significance of miRNA-149 in LC. We also investigated the effect of miRNA-149 on its proliferation, migration, and apoptosis in human LSCC cell line Hep-2.

## 2. Materials and Methods

### 2.1. Tissue Samples

A total of 97 clinical samples were collected from the Department of Otorhinolaryngology, Tianjin Medical University General Hospital, from March 2008 to October 2009. Inclusion criteria were (1) patients who received laryngeal surgery with their pathological type confirmed as squamous cell carcinoma and (2) patients aged 18–70 years. Exclusion criteria were (1) patients with perioperative death, (2) patients who received preoperative radiotherapy or chemotherapy, and (3) patients who conformed to preoperative distant metastasis.

In addition, 46 vocal cord polyp specimens were selected as negative control. All specimens were confirmed by pathological examination. This study was approved by the Ethics Committee of Tianjin Medical University General Hospital, and informed consent was obtained from all patients for sample analysis.

The demographic data of these patients such as gender, age, stage, and smoking and drinking history are recorded in Table 1.

### 2.2. Extraction of Total RNA from Tissue Samples

One hundred mg of LC and vocal cord polyp tissues was prepared. After taking the samples from liquid nitrogen, samples were immediately crushed and moved into a 2 mL sterile Eppendorf tube. Then, 1 mL of Trizol lysis buffer was added to the cells, and the samples were centrifuged at 4°C at 12,000 g for 10 minutes. The upper level was moved to a 1.5 mL EP without RNase. Phenol/chloroform was used for deproteinization, ethanol was used to precipitate RNA, and DEPC water was added to dilute the RNA. Purity of the extracted RNA was tested by an ultraviolet spectrophotometer. The OD260/OD280 ratio value should be greater than 1.8.

### 2.3. qRT-PCR for miRNA-149

Total microRNA was extracted using a microRNA Extraction Kit (miRNeasy Mini Kit, Qiagen, Hamburg, GER). Reverse transcription polymerase chain reaction (qRT-PCR) method was used to measure microRNA-149 levels. Briefly, total microRNA was separated from the extraction kit (centrifugal column type), according to manufacturer's instructions (Tiangen Biotech, Beijing, China). One *μ*g of RNA was traverse transcribed, and quantitative PCR was performed using the ABI 7500 Sequence Detection System (Life Technologies, NY, USA). The expression level of miRNAs was normalized by U6. U6 reverse transcription primers and downstream primers were synthesized by Takara Bio (Kyoto, JP), and the upstream primer was designed and synthesized by Tiangen Biotech (Beijing, China). The objective miRNAs were amplified with the following conditions: 95°C for 30 seconds, 95°C for five seconds, and 60°C for 34 seconds (40 cycles).

The relative expression of microRNA-149 was calculated by methods mentioned by Schmittgen: *F* = 2 − ΔΔCT and ΔΔCT = ctmiRNA-149 − ctmiRNA − U6. CT refers to the number of cycles experienced by fluorescent signals that reached the threshold inside the reactor. All qRT-PCR products were replicated three times.

### 2.4. Cell Culture

We further explored the biological function of miR-149 in LC cell line Hep-2. The human LC cell line Hep-2 was obtained from the American Type Culture Collection (ATCC). Cells were cultured in RPMI 1640 culture medium (Gibco, Paisley, UK) containing 10% fetal bovine serum, 100 u/mL of streptomycin, and 100 *µ*g/mL of penicillin at 37°C with 5% CO_2_.

### 2.5. Transfection

The miR-149 expression plasmid including the vector and control plasmid were purchased from Guangzhou RiboBio Co., Ltd. (Guangzhou, China), and quality control has proven its validity. Cells were inoculated with RPMI 1640 medium without antibiotics the day before the transfection. MiR-149 was diluted, gently mixed with Lipofectamine*™* 2000 (Thermo Fisher Scientific, Waltham, MA, USA), and incubated at room temperature for 20 minutes. The miRNA and Lipofectamine 2000 mixed solution was added into each well that contains the cells and culture medium. Then, the culture plate was gently rocked at 37°C in a CO_2_ incubator and transfected for six hours.

### 2.6. Effect of miR-149 on Human Laryngeal Cancer Cell Proliferation

LC cells were digested by pancreatin, and the cell density was adjusted to 5 × 10^5^/mL. One hundred *µ*L of cell suspension culture was added into each well of the 96-well plate. Then, 10 *µ*L of MTT (5 mg/mL) solution was added into the cell culture chamber and cultured for four hours. Subsequently, 150 *µ*L of DMSO was added to dissolve crystals and oscillated at room temperature for 15 minutes. Cell proliferation was determined through OD values of the cell growth curve at an optical density of 570 nm using a TECAN SpectraFluor plate reader (Tecan, Männedorf, Switzerland).

### 2.7. Flow Cytometry Analysis of Cell Apoptosis

After digestion by pancreatin, 1 mL of cells (5 × 10^5^–1 × 10^6^/mL) was centrifuged at 1,000 rpm for 10 minutes at 4°C, the supernatant was removed, and 1 mL of PBS was added for suspension. Then, 100 *µ*L of binding buffer and 5 *µ*L of annexin VV/7-AAD were sequentially added and cultured for 10 minutes at room temperature. Afterwards, 2.5 *µ*L of actinomycin D (7-AAD) with 150 *µ*L of binding buffer was added to the sequence. Cell apoptosis was analyzed by a fluorescence-activated cell sorter (FACS).

### 2.8. Statistical Analysis

All the experiments were replicated three times in the whole study. GraphPad Prism software (Suite, CA, USA) was used to analyze the relative expression of miR-149. The expression of miR-149 was expressed as the mean and standard deviation (SD), and Mann-Whitney *U* test was used to test the significance of differences. The expression of miR-149 in age, gender distribution, smoking, drinking, and pathological stage in LC patients was compared using Student's test; and survival analysis was performed in LC patients with relative low miR-149 expression (below the mean) and high miR-149 expression (above the mean). The comparison of single-factor analysis of variance and independent *t*-test was used for statistical analysis. *P* < 0.05 was considered statistically significant.

## 3. Results and Discussions

### 3.1. Low Expression of miR-149 in LC Tissues

**Figure 1:**
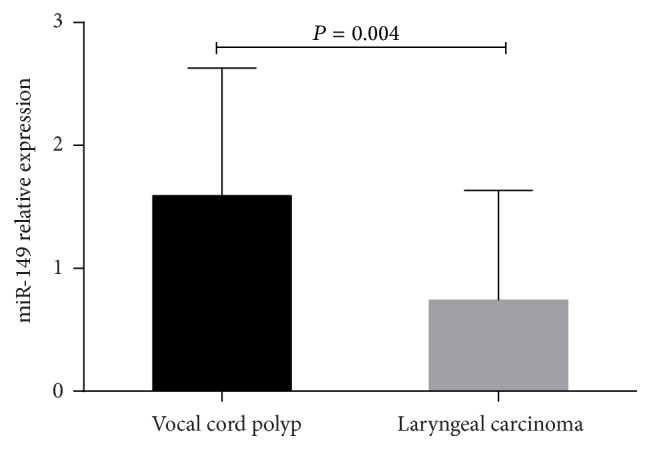
Comparison of miRNA-149 levels in laryngeal carcinoma and vocal cord polyp tissues. Data are shown as mean ± SD and analyzed by Student's *t*-test.

Characteristics (such as age, gender, and smoking status) of the 97 LC patients and 46 controls are shown in Supplemental Table  1 in Supplementary Material available online at http://dx.doi.org/10.1155/2016/8561251. Among the 97 patients with LC, 31 were located in the glottis and 19 were located in the supraglottic region. The age of these patients ranged from 70 to 35 years, with the mean age of 63.8 years. Furthermore, 73 patients were male. Among these patients, 30 (31%) patients had lymph node metastasis, 59 (61%) patients were at stage I or II, and 38 (39%) patients were at stage III or IV. The number of patients with high, middle, and low differentiated squamous cell carcinoma was 23, 49, and 25, respectively. We compared the mean values of miR-149 levels in 97 LC and 46 vocal cord polyp patients by Mann-Whitney *U* test. Results revealed that miR-149 levels in LC tissues were lower than in vocal cord polyp tissues, and the difference was statistically significant (*P* < 0.05, Figure 1).

### 3.2. Low miR-149 Expression Is Correlated with Stages, Differentiation, and Metastasis Situations

The expression of miR-149 in LC patients was further analyzed. Results (Table 1) revealed that the decrease in miR-149 expression was correlated with the stage, differentiation, and metastasis situation of the patients, except for age, gender distribution, and smoking and drinking history.

### 3.3. Survival Analysis

We successfully collected 97 LC samples from patients who received surgery from March 2008 to October 2009, and the end of follow-up time of these patients was October 2014. Among these patients, 17 patients were lost to follow-up, 56 patients survived, and 24 patients died. Among these 24 patients, 17 patients died of tumor recurrence or cervical lymph node metastasis, three patients died of lung metastasis, one patient died of liver metastasis, two patients died of neck tumor invasion of the blood vessels caused by neck bleeding, and one patient died of liver metastasis. Mean miR-149 expression in the 97 patients was 1.46, in which 43 of samples were above the line and 54 of the samples were under the line.

**Figure 2:**
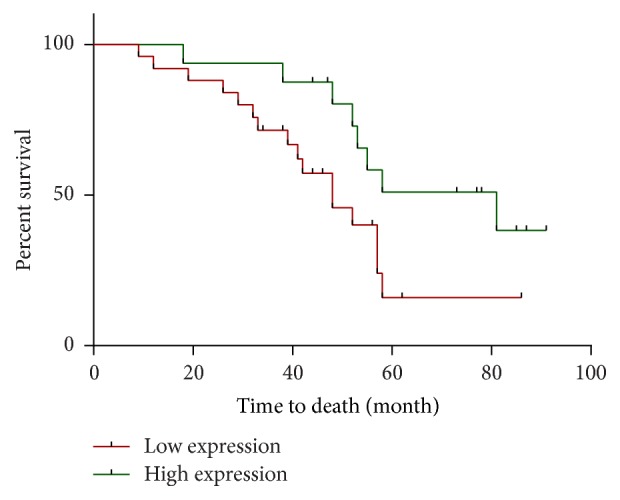
Survival analysis comparison of 97 LC patients with relative low (below mean) and high (above mean) expression of miRNA-149.

Survival analysis revealed the LC patients with low relative expression (median survival of 48 months, 95% CI of ratio 0.2536 to 1.385) had significantly shorter overall survival as compared to patients with a high expression (median survival of 81 months, 95% CI of ratio 0.7222 to 3.3943) (Figure 2, *P* = 0.0405).

### 3.4. Cell Proliferation after miR-149 Transfection

**Figure 3:**
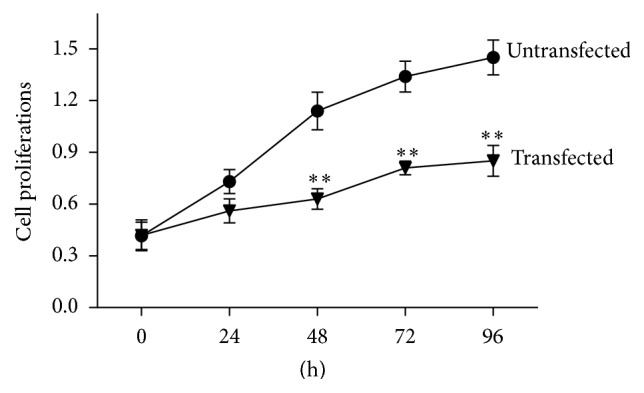
Cell proliferation after transfection of ectopic miRNA-149 to Hep-2 cells; ^*∗∗*^ *P* < 0.05, compared with untransfected cells.

In this study, MTT assay was used to test the effect of the ectopic expression of miR-149 in Hep-2 cells. Cell proliferation was measured at 24, 48, 72, and 96 hours after transfection. Results (Figure 3) revealed that the OD value of transfected cells was significantly lower than in untransfected cells (*P* < 0.05) at 48, 72, and 96 hours. These results suggest that miR-149 inhibits the proliferation of human glioma Hep-2 cells.

### 3.5. Flow Cytometry

**Figure 4:**
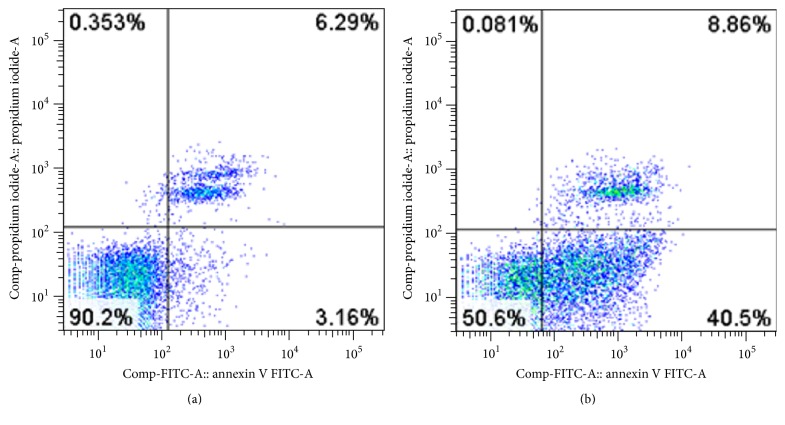
Apoptosis analyzed by flow cytometry after miRNA-149 was transfected into human laryngeal carcinoma cell line Hep-2. (a) Normal Hep-2 cells; (b) Hep-2 cells transfected by miRNA-149.

miR-149 was transfected into human laryngeal carcinoma cell line Hep-2, and apoptosis was detected by flow cytometry 48 hours after transfection. Results revealed that the apoptosis of transfected Hep-2 cells was significantly different from that in untransfected cells: the apoptosis rate of Hep-2 cells was significantly enhanced by miRNA-149 transfection (48.91 ± 3.52% versus 9.8 ± 0.41%, resp.; *P* < 0.05) (Figure 4).

LC is a malignant tumor derived from the epithelial tissues of the larynx. LC occurs more in males between 50 and 70 years [16]. With the development of biotechnology, more molecular markers have been confirmed to be associated with the occurrence of LC, opening up a new gate for the study of the molecular mechanism and development of LC.

miR-149 locates on chromosome 2, and studies have indicated that single nucleotide polymorphisms of miR-149 might be associated with head and neck cancer, as well as the occurrence of pneumoconiosis [17, 18]. Liu et al. [19] found that the combined polymorphism effect of four microRNAs (HSA-146A, HSA-196A, HSA-149, and HSA-499) has a dose-response relationship with head and neck squamous cell carcinoma (HNSCC). This finding implies that the four pre-miRNA binding SNPs (which include HSA-149) may increase the risk of HNSCC.

Tu et al. [20] studied 273 patients with HNSCC and 122 normal healthy controls. Results revealed that the expression of miRNA-149 in HNSCC carcinoma tissues was significantly lower, and that the expression of miRNA-149 could inhibit the migration of HNSCC cells. After further studies, they found that the single nucleotide polymorphism of T/T (pre-miRNA-149) was associated with the expression of miR-149 in HNSCC, while miRNA-149 was determined by miRNA-149 and the prognosis of patients with HNSCC.

In our study, qRT-PCR analysis revealed that the expression of miRNA-149 in LC tissues was significantly lower than that in vocal cord polyp tissues, which is inconsistent with the above reports. Wang et al. [21] indicated that miRNA-149 was epigenetically silenced in colorectal cancer (CRC) and the downregulation of miRNA-149 was associated with the hypermethylation of the neighboring CpG island (CGI). They concluded that, as a methylation-sensitive miRNA, miRNA-149 may play an important role as a tumor suppressor in CRC, which has prognostic and therapeutic implications. Our study shows that miRNA-149 plays a similar role in LC. Thus, we speculate that DNA methylation is also regulated by miRNA-149 methylation in LC tissues.

The survival rate of LC differs in different regions. Survival rate is influenced by many factors such as T stage, N stage, tumor location, and differentiation degree [22–24], while some conclusions were different such as the nutritional status of patients before treatment, smoking and drinking history, and treatment options. Many researchers have indicated that Cox proportional hazards analysis results revealed that T stage, pathological differentiation, and N stage were independent factors that affect the prognosis of LC. In addition, lymph node metastasis was the most important factor that affected survival rate; and five-year survival rate of patients with lymph node metastasis decreased by 50% [25]. We found that LC patients with a low relative expression had significantly shorter overall survival, as compared to those with a high expression. The low expression of miRNA-149 is correlated with stages, differentiation, and metastasis situations, but not with age, gender distribution, and smoking and drinking history. miR-149 analysis results are consistent with the above researches. These results indicate the diagnostic potential of miRNA-149 for LC.

In order to further confirm the correlation between miRNA-149 and the development of LC, we carried out this study of the cell line* in vitro*. These results revealed that miRNA-149 inhibits the proliferation of human Hep-2 cells, and the ectopic expression of miR-149 in Hep-2 cells inhibits proliferation and cell cycle progression. One of the shortcomings of this study is the limited population. In this study, 97 cases of LC tissues were selected for analysis; and this relatively small sample may affect the reliability of the results. Therefore, further expansion of the sample size of this study is necessary.

## 4. Conclusion

In summary, the low expression of miRNA-149 affects the occurrence and development of LC.* In vitro* experiments revealed that miRNA-149 has a significant impact on the proliferation and apoptosis of human Hep-2 cells, and related molecular biology mechanisms need further research.

## Supplementary Material

Supplemental table 1 provides demographic information of vocal cord polyp controls and laryngeal carcinoma patients. The number of vocal cord poly P was 46, and the number of laryngeal carcinoma was 97. The mean age of these two populations was 61.9 ± 12.7 and 63.8 ± 13.4 years old respectively. The gender distribution among these two groups was almost same (P > 0.05). There was significant difference on smoking status between vocal cord polyp and laryngeal carcinoma patients.

## Figures and Tables

**Table 1:** Analysis of miRNA-149 expression in LC patients.

		Case (*n*)	miR-149 level (mean ± SD)	*P* value
Age (year)	<60	46	1.57 ± 0.82	0.479
	≥60	51	1.29 ± 0.74	

Smoking (cigarette/day)	1–20	25	1.62 ± 0.68	0.119
	≥20	36	1.32 ± 0.76	

Drinking (g/day)	<50	45	1.51 ± 1.02	0.053
	≥50	52	1.13 ± 0.89	

Stages	1-2	59	2.37 ± 1.49	0.022
	3-4	38	0.77 ± 0.78	

Lymph node metastasis	None	69	2.45 ± 1.52	0.018
	Yes	28	1.18 ± 1.38	

Pathological differentiation	High	23	0.68 ± 0.74	0.036
	Middle	49	1.23 ± 0.87	
	Low	25	2.48 ± 1.22	
