# Supplementary material for: Clinical Significance of miR-149 in the Survival of Patients with Laryngeal Squamous Cell Carcinoma
Source: Biomed Res Int. 2016 Jun 15;2016:8561251. doi: 10.1155/2016/8561251 (PMC4925956; doi:10.1155/2016/8561251)
Supplement: Supplementary file 1 — Supplemental table 1 provides demographic information of vocal cord polyp controls and laryngeal carcinoma patients. The number of vocal cord poly P was 46, and the number of laryngeal carcinoma was 97. The mean age of these two populations was 61.9 ± 12.7 and 63.8 ± 13.4 years old respectively. The gender distribution among these two groups was almost same (P > 0.05). There was significant difference on smoking status between vocal cord polyp and laryngeal carcinoma patients. [file 8561251.f1.docx]

Supplemental document

Table 1. Demographic information of vocal cord polyp controls and laryngeal carcinoma patients

|  | Vocal cord polyp | Laryngeal carcinoma |
| --- | --- | --- |
| N | 46 | 97 |
| Age (mean ± SD) | 61.9 ±12.7 | 63.8 ± 13.4 |
| Gender (M/F) | 34/12 | 73/24 |
| Smoking status (N/Y) | 19/27 | 61/36 * |

N：non-smoker; Y: with smoking habit; M: male, F: female; *, *P*<0.05, difference in smoking status analyzed by *X^2^*-test.
